# A Review of Occlusion as a Tool to Assess Attentional Demand in Driving

**DOI:** 10.1177/00187208211010953

**Published:** 2021-04-28

**Authors:** Tuomo Kujala, Katja Kircher, Christer Ahlström

**Affiliations:** 1 4168 University of Jyväskylä, Finland; 2 25543 Swedish National Road and Transport Research Institute, Linköping, Sweden

**Keywords:** minimum required attention, visual demand, peripheral vision, self-paced, system-paced

## Abstract

**Objective:**

The aim of this review is to identify how visual occlusion contributes to our understanding of attentional demand and spare visual capacity in driving and the strengths and limitations of the method.

**Background:**

The occlusion technique was developed by John W. Senders to evaluate the attentional demand of driving. Despite its utility, it has been used infrequently in driver attention/inattention research.

**Method:**

Visual occlusion studies in driving published between 1967 and 2020 were reviewed. The focus was on original studies in which the forward visual field was intermittently occluded while the participant was driving.

**Results:**

Occlusion studies have shown that attentional demand varies across situations and drivers and have indicated environmental, situational, and inter-individual factors behind the variability. The occlusion technique complements eye tracking in being able to indicate the temporal requirements for and redundancy in visual information sampling. The proper selection of occlusion settings depends on the target of the research.

**Conclusion:**

Although there are a number of occlusion studies looking at various aspects of attentional demand, we are still only beginning to understand how these demands vary, interact, and covary in naturalistic driving.

**Application:**

The findings of this review have methodological and theoretical implications for human factors research and for the development of distraction monitoring and in-vehicle system testing. Distraction detection algorithms and testing guidelines should consider the variability in drivers’ situational and individual spare visual capacity.

## Introduction

The visual occlusion technique was developed by Senders et al. (1967) to evaluate attentional demand in driving. By intermittently blocking (i.e., occluding) the driver’s line of sight, for example with an occlusion visor or opaque glasses, the (visual) attentional demand can be estimated as the fraction of time that the driver’s visual field is *unoccluded*. The occlusion technique can also be used to evaluate the analog but opposing concept of “spare visual capacity,” which measures the fraction of time that the driver’s visual field is *occluded* (Safford, 1971). These fractions can inform about the required (visual) information sampling frequency in driving. This understanding is important for defining safe attention allocation behaviors in traffic for scientific, engineering and regulatory purposes.

Both attentional demand and spare visual capacity, as measured via visual occlusion, must be coupled with an assessment of successful driving performance. In the experiments of Senders et al. (1967), the drivers themselves determined what was to be considered as successful driving. The drivers’ visual field was occluded by default, meaning that they were essentially driving while blindfolded. However, as soon as the uncertainty about their own position in relation to the road became too high, the drivers could voluntarily unocclude their vision to recalibrate their mental model of the surroundings. The attentional demand of the situation was then estimated as the frequency of requested viewing instances. This is an example of a *self-paced* visual occlusion experiment. Senders et al. (1967) also did experiments with fixed occlusion and unocclusion times (i.e., *system-paced* visual occlusion) to investigate how fast the drivers were willing to drive with intermittently occluded vision. The outcome of this series of experiments provided support for the assumption that the driver’s attention needs to be only intermittently directed to the road and that the attentional demand varies with speed and road curvature.

**Figure 1:**
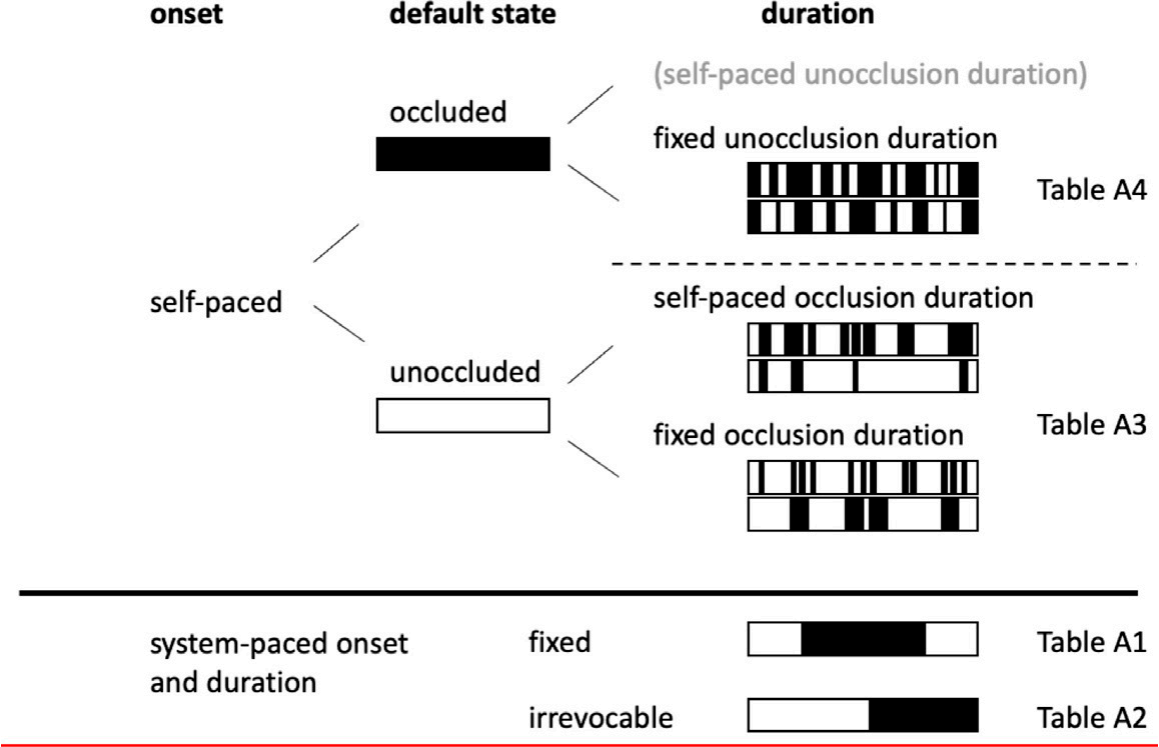
Key parameters for an occlusion experiment. Onset describes if the occlusion is controlled by the driver (self-paced) or the experimenter (system-paced). Default state describes if vision is occluded or unoccluded by default. Duration illustrates the types of the unocclusion/occlusion duration (self-paced, fixed or irrevocable). Black bars illustrate occluded vision. Relevant studies of each category are listed in the referred Tables in Appendix.

As already indicated, there are several free parameters in the setup of a visual occlusion experiment (Lansdown et al., 2004), as can be seen in Figure 1. The main difference between these settings lies in how much freedom the participant is given in controlling when and for how long the visual field is occluded. In a *system-paced onset*, the experimenter decides when, where, and for how long to occlude, including the special case of irrevocable occlusion. For safety reasons, this setting has mostly been used in simulators and on closed roads. In system-paced occlusion, the location and duration of the occlusion are used as independent variables to assess how the withheld information affects driving performance or other relevant capacity (e.g., hazard perception). A *self-paced onset*, on the other hand, allows the driver to decide when to occlude or unocclude, depending on whether the default state of the apparatus is “unoccluded” or “occluded.” With an *unoccluded default state*, which has mostly been used in real traffic, drivers plan ahead and indicate with the activation of the occlusion when and where they do not need any visual information for the driving task at hand. An *occluded default state* requires the driver to activate access to visual information and has been more common in research done in simulators and on closed roads. The *duration of the occluded period* can be self-paced or fixed. If the occlusion duration is self-paced, drivers can choose freely when to terminate the occlusion depending on their level of uncertainty. In the self-paced onset setting, the choice to (un)occlude and, if self-paced as well, the occlusion time (or distance) are typically treated as dependent variables whose variation with external factors is of interest. The various settings represent different approaches to investigating spare visual capacity.

With this review, we intend to assess how visual occlusion studies with various settings have contributed to the understanding of attentional demand and spare visual capacity in driving, thereby illustrating what the method can achieve while also pointing out its limitations. Based on this background, we then discuss how some of the weaknesses can be mitigated, for instance by triangulation with other methods. This can help future research go beyond the identification of spare visual capacity to a fuller understanding of the attentional demands in driving.

## Method

A literature search for the term “‘visual occlusion’ driving” was carried out using Google Scholar. The search found 2130 results (patents not included), which were sorted by relevance as defined by Google Scholar (https://scholar.google.com/intl/en/scholar/about.html). The inclusion criteria for the selected studies were that (1) the forward visual field was temporally (as opposed to spatially) occluded (i.e., visually interrupted) while the participant was driving and (2) the studies had to assess the visual demands of driving. The search results were reviewed for title and excerpt of the articles, and for the abstract or full-text articles if needed, and excluded if they did not fulfill the inclusion criteria. Studies where the participant was watching images or videos of driving were excluded, as were papers where the participant was a passenger in a moving vehicle. Furthermore, a number of articles related to in-vehicle information system testing (e.g., Foley, 2008; Gelau et al., 2009; Lansdown et al., 2004) were excluded as these evaluations are usually carried out while standing still and the objective of this line of research is not to assess the visual demands of driving but rather to focus on the demands of in-car tasks. Relevant references in the found articles (five) were also included in the review. In screening phase, 2046 search results were excluded. In total, 89 full-text articles were assessed for eligibility, of which 37 articles were excluded for not fulfilling the inclusion criteria. As a result, 57 studies in 52 publications were included in the qualitative synthesis.

## Results

**Figure 2:**
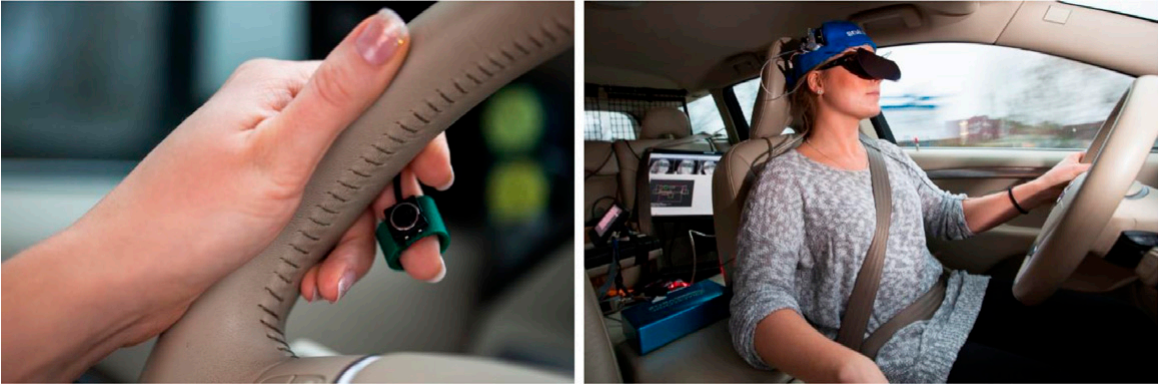
An example of a visual occlusion mechanism that is activated by a micro-switch placed on the middle finger from the on-road study by Kircher et al. (2020).

A summary of the 57 included studies reported in 52 publications is provided in Tables A1–A4 (Appendix). For each study, the main objective of the experiment, the number of participants, the driving environment (motorway, test track, or driving simulator), and the settings of the occlusion experiment are provided. The latter also includes the mechanisms used to operate the occlusion device, ranging from finger or foot operated switches/pedals (Figure 2) to simply closing one’s eyes, and the occlusion area, which can consist of the entire visual field or its central parts. The tables are divided based on the occlusion settings that are used (Figure 1), with system-paced studies in Table A1, system-paced with irrevocable occlusion in Table A2, self-paced studies with an unoccluded default setting in Table A3, and self-paced studies with an occluded default setting in Table A4. The remainder of the results section provides an overview of what the reviewed visual occlusion studies have taught us about how to measure spare visual capacity in driving and what we have learned about it.

### Benchmarking Spare Visual Capacity

Visual occlusions provide an estimate of spare visual capacity, but there are no obvious criteria stating if an observed occlusion frequency or duration indicates operation below, at, or above capacity. Anderson et al. (2000) assumed that self-paced occlusion is likely an underestimate of the actual spare capacity. Accordingly, many participants in Kircher et al. (2020), who used the self-paced setting, reported that they had occluded below their perceived maximum capacity, hence keeping a safety margin. An incident or collision caused by the driver during an occlusion would indicate that the minimum required information had not been sampled, but the absence of such occurrences is no guarantee for sufficient sampling.

To get a measure that is more indicative of true capacity in self-paced settings for a part-task of driving, such as steering, time-to-line-crossing (TLC) at the end of the occlusion (TLC_end_) can be added to the occlusion time (OT), providing an estimate of the total time from occlusion onset until the vehicle leaves the lane (Godthelp, Milgram, et al., 1984). Whereas the OT + TLC_end_ metric describes steering performance, similar integrative metrics can be used to describe thresholds of spare visual capacity in other dynamic part-tasks of driving (e.g., time-to-collision [TTC] in longitudinal control tasks; van der Horst, 1990). These metrics apply only to the lane-keeping and distance-keeping tasks in repeated measurements in controlled conditions. How to combine such separate models in more naturalistic driving situations with multiple concurrent demands is not known, but computational modeling and simulation of the interactions of the part-tasks’ competing visual demands may be a way forward (Jokinen et al., 2020). The question is whether it will be possible to move from “capacity to stay on the road” in controlled environments to “minimum required attention” in more complex driving environments with multiple actors and competing demands.

Given the current lack of objective benchmarks, an important question is whether visual occlusion is a solid empiric correlate with respect to the construct of spare visual capacity. As reviewed in the next subsection, self-paced occlusion studies have shown systematic and expected variations in occlusion times based on the manipulations of factors such as speed, road curvature, road environment, lane width, distance to other traffic, and type of maneuver. Although there are large individual differences in the effects of these variables and the occlusion times, the mean effects of increasing complexity indicate lower occlusion times across the studies. This is in line with the presumption that occlusion time has a correlation with spare visual capacity. Accordingly, system-paced studies have shown that drivers are able to maintain an acceptable level of performance while occluded for considerable time periods. An upper limit of spare visual capacity can be attained in a system-paced occlusion setting by gradually increasing the occlusion time until performance failures occur (e.g., Anderson et al., 2000). Studies with irrevocable occlusion have shown that drivers can refrain from crashing in a simulated motorway curve with surrounding traffic for, on average, about 7 s (Kircher et al., 2018), and on a straight road they stayed within a field of approximately five meters in width for almost 8 s (Zwahlen & Balasubramanian, 1974).

### Spare Visual Capacity Varies With Situational Demands

In spite of the lack of objective benchmarks, occlusion studies have shown direct evidence that there can be spare visual capacity in driving (Anderson et al., 2000; Kircher et al., 2020; Senders et al., 1967) and that occlusion times vary according to the expected effects of situational demands. The amount of available spare capacity depends on several factors. Under otherwise similar circumstances, drivers choose to occlude their vision for shorter percentages of time when driving at higher speeds (Courage et al., 2000; Farber & Gallagher, 1972; Godthelp et al., 1984; Mourant & Ge, 1997; Senders et al., 1967), with narrower lane widths (Courage et al., 2000; Farber & Gallagher, 1972; Godthelp et al., 1984; Mourant & Ge, 1997; Senders et al., 1967; van der Horst & Godthelp, 1989), when driving in sharper curves (Anderson et al., 2000; Backs et al., 2003; Courage et al., 2000; Senders et al., 1967; Tsimhoni & Green, 2001; Wooldridge et al., 1999) or on more complex road geometries (Easa & Ganguly, 2005; Easa & He, 2006). Many of the reviewed studies, especially in the system-paced category, attempt to isolate the effect of a single external factor on one behavioral aspect, with a special focus on either longitudinal or lateral vehicle control. There is a risk that this approach misses interaction effects that can occur if the driver must deal with multiple simultaneous requirements.

However, studies that investigated the tactical driving level (Michon, 1985) indicate that variations in more complex conditions also influence the possibility for self-paced occlusion (Kircher & Ahlström, 2018; Liu et al., 2020; Mourant & Mourant, 1979; Steele & Gillespie, 2001). Kircher and Ahlström (2018) found that maneuvers that require interaction with other traffic, like a lane change into the fast lane, lead to fewer and shorter occlusions than driving in the slow lane. Motorway driving has been found to afford more occlusion time than urban or rural driving, in spite of higher speeds on the motorway (Liu et al., 2020; also occlusion distance: Kujala et al., 2016). In heterogeneous environments, drivers often choose to occlude their vision in relation to external circumstances, such as obstacles in the infrastructure, the distance to other traffic and interactions between several such factors (Kircher et al., 2020, Liu et al., 2020; Pekkanen et al., 2017, 2018; Steele & Gillespie, 2001). In general, the findings suggest that a lower complexity of the driving scenario or maneuver increases spare visual capacity.

With increasing levels of vehicle automation, driver assistance systems have also been found to reduce the need for visual sampling. For example, visual demand is reduced when driving with adaptive cruise control (Hoedemaker & Kopf, 2001) or with lane keeping assistance (de Vos & Godthelp, 1999; Griffiths & Gillespie, 2005; Mars et al., 2014; Steele & Gillespie, 2001). The downside of this reduced visual demand is that the driver may be inclined to be visually and mentally distracted from the supervision of the systems’ behaviors and the traffic environment by secondary activities. Only a handful of studies have assessed how additional cognitive load under occlusion affects the visual demands of driving and drivers’ ability to predict the development of the traffic scenario. Monk and Kidd (2008) found an improvement in lane tracking under cognitive load during occlusion, whereas hazard perception performance worsened during a concurrent visual task (Borowsky et al., 2015, 2016; Samuel & Fisher, 2015).

### Inter-Individual Differences in Spare Visual Capacity

Already Senders et al. (1967) speculated on and observed with small sample sizes inter-individual differences in drivers’ capacity to occlude the driving view in similar traffic conditions. Individual differences have been shown in many occlusion studies, but the main evidence of large individual variations in spare visual capacity comes from self-paced studies. For instance, Liu et al. (2020) showed that spare visual capacity was highly dependent on undetermined individual factors in different on-road traffic scenarios. However, occlusion studies have also succeeded in revealing some of the factors that affect this individual capacity.

One factor that has been shown to affect occlusion behavior is age. Older drivers require significantly more visual information in motorway driving (Mourant & Mourant, 1979; Rackoff, 1975) and in curve driving with various curvatures (Tsimhoni & Green, 1999). One explanation could be that older drivers, on average, require more time to process the available visual information and are less effective in their visual search patterns (Shinar et al., 1978). In all these studies, some aged drivers performed at the same level as the younger drivers, indicating that aging does not lead to similar effects for all drivers. Furthermore, average occlusion distance in self-paced occlusions has not been found to vary significantly with age (Kujala et al., 2016), and neither has TTC assessments under occlusion (e.g., Kiefer et al., 2006).

A related factor is driving experience. When predicting occlusion times based on a supervisory control model, Blaauw et al. (1984) found that the predictions underestimated inexperienced drivers’ actual occlusion times, but not those of experienced drivers. This finding suggests that a different control model may be more suitable for inexperienced drivers. Based on the finding that experienced drivers can execute simple steering maneuvers while intermittently occluded, Cavallo et al. (1988) suggested that experienced drivers are more proactive when it comes to path control in curve driving, whereas novice drivers are more reactive. This is also supported by findings that experienced drivers use more complex and dynamic visual sampling strategies and have a better ability to estimate and predict the vehicle’s position during occlusions (Chen, 2013). That experienced drivers are more capable of constructing useful predictions is also indicated by longer self-paced occlusion times for comparable levels of driving performance and more adaptation of occlusion times when occluded during driving-related multitasking and foggy or night-time driving conditions (Blaauw, 1984). A similar finding was made by Kujala et al. (2016) where the more experienced drivers were able to achieve more accurate lane-keeping performance than the least experienced drivers with similar occlusion distances.

Senders et al. (1967) attributed inter-individual differences to information-forgetting rate and the maximum level of tolerated uncertainty during occlusion, and also to how far ahead the driver samples information. The ability to make use of the sampled information and predict what is happening during the occlusion is yet another factor, which depends on the individual’s ability to extract information during the preceding sampling (Shinar et al., 1978) and on its duration (Chen & Milgram, 2011). Individual differences can also be attributed to preferred safety margins (Kujala et al., 2016; Pekkanen et al., 2017), in terms of how drivers try to maintain information redundancy during sampling (Milgram, 1983; Milgram et al., 1982) or task difficulty (Pekkanen et al., 2017) at an individually comfortable level. Instead of overestimations, it seems that drivers tend to underestimate TLC (Godthelp 1984), TTC (Kiefer et al., 2006), and distances (Saffarian et al., 2015) while occluded. The drivers in the study by Godthelp et al. (1984) preferred to sample, on average, at about 40% of the available time before estimated lane crossing.

## Discussion

Visual occlusion in its varying forms has been used as a tool to establish the minimum visual information input necessary for driving in a foresighted and controlled manner. Occlusion studies have provided evidence of spare visual capacity in driving and support for open-loop (i.e., intermittent) control models of driving performance (e.g., Godthelp et al., 1984). There is convincing evidence that spare visual capacity relates to what we can summarize as the predictability of the situation, which is dependent on a combination of factors like the infrastructure, other traffic, and one’s own capabilities and maneuvering intentions.

This qualitative literature review provided an overview of the central findings in the occlusion literature, but meta-analysis—where applicable—would be the next logical step to acquire a more detailed quantitative understanding about the general effects across studies. However, due to the disparity of experimental designs and targets of research (Tables in Appendix) and the fact that the data are often not reported or open access in a manner that would enable a meaningful meta-analysis, this step might be difficult if not impossible.

### Limitations

Visual occlusion is a flexible method that gives access to insights about the availability of spare visual capacity and about the occasions when visual input is needed. However, as outlined in the benchmark section, the lack of criteria that determines the objectively available spare visual capacity is a fundamental challenge.

Visual occlusion can be seen as a coarse method given its time scale (as compared to fixations) and in that it blocks the entire visual field of the driver. In some studies, a small peripheral area was left intact (Borowsky et al., 2015, 2016; Kircher et al., 2020, Liu et al., 2020; Pekkanen et al., 2017, 2018; Samuel & Fisher, 2015). This means that occlusion studies do not allow an assessment of which specific fields or objects are most crucial for the driver in each situation. A combination of occlusion with eye tracking can indicate the foveal targets in the unoccluded periods (e.g., Anderson et al., 2000; Borowsky et al., 2016; Kircher et al., 2020) but cannot determine the degree of importance of peripheral information. Partial occlusion (e.g., foveal vision only, peripheral vision only, or gaze-contingent windows where nonfoveal fields are occluded based on real-time eye tracking) can provide hints for how the different regions are used and what their relative importance is for part-tasks of driving, depending on how performance degrades or sampling changes if parts of the visual field are taken away (e.g., Gordon, 1966; Wood & Troutbeck, 1994). Yet, removing part of the visual field may lead to qualitatively altered sampling strategies. Therefore, a direct estimation of what information is normally sampled with foveal or peripheral vision may not be possible with partial occlusion.

Self-paced occlusion with an unoccluded default state resembles the situation where a driver chooses to execute an additional visual task while driving. However, in most studies, visual occlusion merely blanks out visual input, whereas additional visual tasks also require some mental focus but do not necessarily take away peripheral vision. Visual occlusion lets the driver focus on the latest impression of the scene, providing the possibility to make predictions about its likely development. Very few studies have investigated whether an additional task during visual occlusion hampers the driver’s prediction abilities (Borowsky et al., 2015, 2016; Monk & Kidd, 2008; Samuel & Fisher, 2015). More studies on the topic are needed for a thorough differentiation of the effects of merely blocking the driver’s visual field versus additional task execution that also involves mentally focusing on something else.

Visual occlusion is rather obtrusive and, especially in its self-paced version, can put mental load on the driver. For example, in some of ’s (1967) experiments, the participants were given a break after 15 min because “the task of driving was an arduous one,” and comments from participants in our own studies indicate that occlusion experiments are experienced as fatiguing. Also, blocking a driver’s vision outside simulators can be perceived as ethically problematic (Anderson et al., 2000; Tsimhoni & Green, 1999), or as Senders et al. (1967) put it, “perhaps a little risky.” This could be one reason behind the rareness of on-road studies employing the method. Using vehicles equipped with dual control and experienced safety drivers that are ready to intervene, as used in Kircher et al. (2020), is a relevant safety procedure in on-road studies in actual traffic. Differences between simulator and on-road studies were outside the scope of the current review, but it should be noted that there might be variations in the outcomes depending on the ecological validity of the driving task.

### Occlusion and Eye Tracking

Historically, studies using visual occlusion and studies using eye tracking have largely proceeded independently of each other, with the former mainly focusing on when visual information was not needed for driving and the latter categorizing what was sampled without really considering its necessity. As such, the two methods complement each other, and much can be gained by combining them. However, this is rarely done, and only a handful of studies have combined the techniques (see notes in Tables A1–A4, Appendix). Instead, eye tracking has gradually become the de facto standard in driver attention research.

Mobile eye trackers were first introduced to traffic research around the seventies (Mourant & Rockwell, 1970, 1972; Rockwell et al., 1968). In these early days of eye-tracking research, the focus was on understanding the eye movement patterns of experienced, accident-free drivers. The importance of peripheral vision was still acknowledged and deduced by specifically considering which targets were not fixated foveally. Technological advancements not only led to a more frequent and widespread use of eye tracking, but also saw a shift in how the data were interpreted. It became more common to only consider the foveally fixated targets, listing and categorizing them and labeling them as “relevant for driving” or not (Crundall et al., 2006; Garrison & Williams, 2013). This analysis of foveal fixations on specific targets is appealing for its putative simplicity; however, it comes at the risk of neglecting what cannot be easily observed—namely, the information sampled via peripheral vision (Rosenholtz, 2016; Wolfe et al., 2019) and the determination of the necessary amount of information for a task (Kircher & Ahlström, 2017). Gradually, the advent of mobile eye trackers directed the focus of research on attention in traffic toward the targets that drivers foveally focus upon, and to the conclusion that drivers are inattentive as soon as they glance at targets deemed “not relevant for driving” (Garrison, 2011) without considering any possibly available spare visual capacity.

As compared to occlusion, eye tracking alone cannot reveal redundancy in fixations at targets, that is, which of the fixations were actually needed for gathering the required information (Kircher et al., 2020). Eye tracking delivers insights about specific (foveal) gaze targets (e.g., pedestrian approaching a crosswalk), while occlusion makes sure that no redundant information is unjustly assumed to be relevant (e.g., repeated fixations on an empty road). Capitalizing on this, it has been shown that there are redundant glances to the forward roadway in normal driving (Anderson et al., 2000; Kircher et al., 2020), but also necessary maneuver-dependent glances off the forward roadway (e.g., on mirrors; Kircher et al., 2020).

### Minimum Required Attention and Future Work

Chen and Milgram (2013) argue that instead of gross metrics, the focus in occlusion research should be on the situational and individual variability of the information sampling. There are large situational and individual differences in occlusion times in similar scenarios without explanation (e.g., Kujala et al., 2016). This also means the lack of objective benchmarks. However, we are not aware of a more objective method than occlusion for assessing spare visual capacity in dynamic tasks. This capacity always has a subjective component, even if the capacity can be argued to decrease (on average) with the increasing complexity or unpredictability of a scenario.

For a deeper understanding of the minimum amount of information that needs to be sampled for attentive driving in real traffic scenarios, and thereby going beyond single-factor control models while attempting to preserve access to individual factors, we suggest combining the rather data-driven approach of visual occlusion with a theory that identifies relevant information a priori. In this context, visual occlusion, possibly in combination with other methods like eye tracking and think-aloud, could indicate how frequently the needed information is sampled and whether this is done foveally or peripherally. The Minimum Required Attention (MiRA) theory (Kircher & Ahlström, 2017) could be used as a starting point for defining the attentional requirements, at least with respect to so-called static requirements, which are related to infrastructure and traffic regulations. Self-paced occlusion frequency, duration, and location can be observed in relation to a systematic combination and variation of requirements, and system-paced occlusion of predefined aspects can increase the precision of the findings. To establish sampling requirements in dynamic situations, factors like surrounding traffic can be varied while controlling other factors, to assess the impact on occlusion possibilities. Computational modeling may prove to be useful for simulating the dynamic requirements and interactions of multiple demands (compare Jokinen et al., 2020). Equally important as knowing the situational targets is knowing how the situational factors affect and interplay with the required information sampling frequencies of these targets. Existing self-paced occlusion studies have shown how drivers experience these frequencies, but more advanced tools are needed to evaluate the validity of these assessments in complex scenarios.

### Implications for Human Factors Research and Practice

What is essential for minimum required attention, as defined above, is the timing of information sampling in open-loop driving with various competing demands (compare Godthelp et al., 1984). The definition of minimum required attention differs from the definition of visual demand used by, for example, Backs et al. (2003) and Tsimhoni and Green (1999 and 2001), who operationalized it as unoccluded time divided by total time within a segment of interest. As a gross measure, similar to the percentage of unoccluded time per drive by Mourant and Ge (1997), it loses the situational information on the timing of the demands. Similarly, driver distraction guidelines (Young & Zhang, 2015) based on system-paced occlusion testing for in-car tasks neglect one of the most important factors of spare visual capacity, that is, the driver’s ability to time the in-car glances in accordance with the variable visual demands of driving. As such, these methods seem to evaluate only the visual demands of the in-car task and not its compatibility with driving.

The observed variability in spare visual capacity in driving casts doubts on general (off-forward) glance duration thresholds for distraction monitoring and visual distraction testing of in-car tasks (e.g., Young & Zhang, 2015). When testing the distraction potential of in-car devices or tasks, the individual differences in spare visual capacity should be considered and controlled in order to provide reliable results (Broström et al., 2016). Furthermore, the presented evidence suggests that acceptance of an in-car task to be conducted while driving cannot be judged only by its effects on visual sampling in an isolated part-task of driving. On the other hand, the acceptance thresholds might be too strict if the driver’s capacity to utilize peripheral vision in the task is not considered (e.g., as in lane keeping). Even more importantly, the thresholds might be too low if the visual and cognitive demands of the in-car task interfere with the demands of such part-tasks of driving that have not been evaluated (e.g., hazard perception; Borowsky et al., 2014).

## Conclusions

The occlusion technique can complement eye tracking in studies on the attentional demand of driving by indicating the driver’s spare visual capacity. Occlusion studies have shown that spare visual capacity varies across situations and drivers and have indicated environmental, situational, and inter-individual factors behind the variability. The level of understanding that can be achieved with the technique depends on the selection of the occlusion method (self-paced vs. system-paced).

The findings of this review have methodological implications for human factors research and practical applications for the development of distraction monitoring and in-vehicle system testing. Distraction detection algorithms and testing guidelines need to consider the variability in situational and individual spare visual capacity. Oversimplifying the attentional demand of driving should be avoided in order to make valid and reliable conclusions on whether a driver is distracted or not. While there are a number of occlusion studies looking at various aspects of attentional demand, we are still only beginning to understand how the demands vary, interact and covary. Triangulation of various methods together with occlusion may be required for this inquiry.

## Key Points

- Spare visual capacity varies with situation and driver.
- Combining eye tracking with the occlusion technique can enable the indication of requirements for and redundancy in visual information sampling.
- The appropriate occlusion setting depends on the target of the research.
- Distraction monitoring and testing needs to consider the variability in attentional demand.
- We are still only beginning to understand how attentional demands of driving vary, interact, and covary.

## Appendix

**Table A1:** Studies Using System-Paced Occlusion

Reference	Occlusion Method	Driving Environment	*N*	Objective
Senders et al. (1967), setup I	system-paced onset, fixed open duration (.25 s, .5 s, 1.0 s) and occlusion duration (0.5 s - 9.0 s), total field of view	open motorway and test track	5	estimate how attentional demand vary with combinations of road, vehicle, and speed
Godthelp (1985), experiment I	system-paced onset in relation to overtaking maneuver, fixed occlusion duration (.1 s, 1.0 s), total field on screen	sim fixed base	9	differences in open- and closed-loop steering in a lane-change maneuver
Godthelp (1985), experiment II	system-paced onset in relation to overtaking maneuver, fixed occlusion duration (3.0 s), partial field with far periphery open	closed motorway	7	differences in open- and closed-loop steering in a lane-change maneuver
Godthelp (1986)	system-paced onset in relation to curve, occlusion duration (1.5 s, 1.8 s), total field of view	closed motorway	6	ability to generate anticipatory steering actions
Cavallo et al. (1988)	system-paced onset in relation to curve (2 s before, at start, middle, or near the end), fixed occlusion distance, total field of view	test track	20	influence of driving experience on anticipation and performance in negotiating curves while occluded
van der Horst (1990)	system-paced onset, fixed stroboscopic open duration (.1 s in 5 or 25 Hz), total field of view	closed road	12	importance of optic flow versus speed and distance when determining TTC
Anderson et al. (2000), study II	system-paced onset, fixed open duration (.5 s) and gradually increasing occlusion duration until failure, total field of view	test track	24	assess driver tolerance for increased workload imposed by roadway geometric features
Hildreth et al. (2000)	system-paced onset, fixed occlusion duration (1.0–4.0 s), total field on screen	sim fixed base	6	steering behavior with or without occlusion
Kiefer et al. (2006)	system-paced onset in relation to object, fixed open duration (1.0 s), total field	test track	51	time to collision judgment under realistic rear-end crash scenario conditions
Lee et al. (2007)	system-paced onset in relation to surrounding vehicles, fixed occlusion duration (1.0 s), total field on screen	sim fixed base	12	hazard detection based on cognitive load and previous occlusion
Monk and Kidd (2008)	system-paced onset in relation to vehicle manipulation, fixed occlusion duration (1.0 s), total field on screen	sim fixed base desktop	20	lane drift recovery after occlusions with and without cognitive loads
Borowsky et al. (2015) ^a^	system-paced onset in relation to hazard, fixed occlusion duration (2.0 s), central field on screen	sim fixed base	12	hazard detection during visual interruption with additional task
Borowsky et al. (2014) ^a^	system-paced onset in relation to hazard, fixed occlusion duration (2.0 s), central field on screen	sim fixed base	54	hazard detection performance depending on occlusion type
Johns and Cole (2015)	system-paced onset, fixed open duration (.4 s) and occlusion duration (2.5 s), total field of view	sim fixed base	6	model of intermittent compensatory steering control
Samuel and Fisher (2015) ^a^	system-paced onset, fixed occlusion duration (2.0 s) and open duration (1.0 s, 2.0 s, 3.0 s, 4.0 s), central field on screen	sim fixed base	45	minimum forward roadway glance duration
Borowsky et al. (2016) ^a^	system-paced onset in relation to hazard, fixed occlusion duration (2.0 s), central field on screen	sim fixed base	54	hazard detection performance depending on occlusion type

*Note*. ^a^Eye- tracking was used simultaneously with occlusion.

**Table A2:** Studies Using System-Paced Irrevocable Occlusion

Reference	Occlusion Method	Driving Environment	*N*	Objective
Zwahlen and Balasubramanian (1974)	system-paced onset, total field of view	test track	2	ability to maintain a straight path when driving with closed eyes
Zwahlen and DeBald (1986)	system-paced onset, total field of view	test track	12	ability to maintain a straight path when driving with closed eyes
Kaptein et al. (1996)	system-paced onset in relation to object, total field on screen	sim fixed base	13	assess capability to target-brake under occlusion as compared to open
Wallis et al. (2007)	system-paced onset in relation to lane change, total field on screen	sim motion base	20	investigate vehicle steering control in lane changing and the role of different sources of sensory feedback
Saffarian et al. (2015)	system-paced onset in relation to object, total field on screen	sim fixed base	24	assess capability to target-brake under occlusion as compared to open
Kircher et al. (2018) ^a^	system-paced onset in relation to curve, total field on screen	sim moving base	22	mental model of traffic and behavior under irrevocable occlusion

*Note*. ^a^Eye- tracking was used simultaneously with occlusion.

**Table A3:** Studies Using Self-Paced Occlusion With an Unoccluded Default Setting

Reference	Occlusion Method	Driving Environment	*N*	Objective
Safford (1971) ^a^	self-paced onset, self-paced occlusion duration, total field of view	open road	6	spare visual capacity assessment (also when intoxicated with CO)
Rackoff (1975) ^a^	self-paced onset, self-paced occlusion duration, total field of view	open motorway	10	visual search and self-paced occlusion in old and young drivers
Shinar et al. (1978)	self-paced onset, self-paced occlusion duration, total field of view	open motorway	15	relation between information processing tasks and ability to occlude
Mourant and Mourant (1979) ^a^	self-paced onset, self-paced occlusion duration, total field of view	open motorway	10	visual search and self-paced occlusion in old and young drivers
Tsimhoni and Green (2001)	self-paced onset, fixed occlusion duration (.5 s), total field on screen	sim fixed base	16	impact of visual demand on secondary task interactions
Kircher and Ahlström (2018) ^a^	self-paced onset, self-paced occlusion duration, partial field with far periphery open	open motorway	12	evaluation of methods for attention assessment
Kircher et al. (2020) ^a^	self-paced onset, self-paced occlusion duration, partial field with far periphery open	open motorway	25	differences between necessary and unnecessary glances away from the forward roadway
Liu et al. (2020)	self-paced onset, fixed occlusion duration (1.0 s, 1.4 s, 1.8 s, 2.2 s, 2.6 s), partial field on screen with far periphery open	sim fixed base	30	assess attentional demand of different contextual factors

*Note*. ^a^Eye- tracking was used simultaneously with occlusion.

**Table A4:** Studies Using Self-Paced Occlusion With an Occluded Default Setting

Reference	Occlusion Method	Driving Environment	*N*	Objective
Senders et al. (1967), setup II	self-paced onset, fixed open duration (.5 s), total field of view	open motorway and test track	5	estimate how attentional demand vary with combinations of road, vehicle, and speed
Farber and Gallagher (1972)	self-paced onset, fixed open duration (.5 s), total field of view	closed road	6	effect of degraded visibility conditions on steering and control tasks
Hicks and Wierwille (1979)	self-paced onset, fixed open duration (.2 s), total field on screen	sim motion base	30	assessment of methods to detect changes in driver workload
Godthelp et al. (1984)	self-paced onset, fixed open duration (.55 s), total field of view	closed motorway	6	relationship of TLC to occlusion and TLC as driving performance measure
Blaauw et al. (1984)	self-paced onset, fixed open duration (.55 s), total field of view	closed motorway	12	supervisory role of the driver in lateral position control
Godthelp and Käuppler (1988)	self-paced onset, fixed open duration (.55 s), total field of view	closed motorway	6	using TLC for improved understanding of the relation between vehicle handling, lateral control and occlusion
Krammes et al. (1995)	self-paced onset, fixed open duration (.5 s), total field of view	test track	55	visual demand in relation to curve radius
Mourant and Ge (1997)	self-paced onset, presumably self-paced duration, total field on screen	sim fixed base VR	8	quantification of attentional demand as function of speed, curvature, oncoming traffic
de Vos and Godthelp (1999)	self-paced onset, fixed open duration, total field of view	closed motorway	8	reduced visual demands with lane keeping support
Tsimhoni and Green (1999) ^a^	self-paced onset, fixed open duration (.5 s), total field on screen	sim fixed base	24	visual demand of driving in curves with different radii
Anderson et al. (2000), study I	self-paced onset, fixed open duration (.5 s), total field of view	open motorway and test track	24	assess driver workload imposed by roadway geometric features
Anderson et al. (2000), study III^a^	self-paced onset, fixed open duration (.5 s), total field on screen	sim fixed base	24	simulator validity with respect to visual demand in curves
Courage et al. (2000)	self-paced onset, fixed open duration (.5 s), total field of view	sim fixed base	10	differences in attentional demand due to speed, curvature, lane width
Wooldridge et al. (2000)	self-paced onset, fixed open duration (.5 s), total field of view	open motorway and test track	24	assess driver workload imposed by roadway geometric features
Hoedemaker and Kopf (2001)	self-paced onset, fixed open duration (.5 s), central field of view	test track	24	changes in visual demand with adaptive cruise control
Steele and Gillespie (2001)	self-paced onset, fixed open duration (1.0 s), total field on screen	sim fixed base desktop	22	reduced visual demand with haptic lateral guidance
Backs et al. (2003)	self-paced onset, fixed open duration (.5 s), total field on screen	sim fixed base	15	relate visual demand with cardiac measures of workload
Easa and Ganguly (2005)	self-paced onset, fixed open duration (.5 s), total field of view	sim fixed base desktop	9	effects of complex horizontal highway alignments on visual demand
Griffiths and Gillespie (2005)	self-paced onset, fixed open duration (1.0 s), total field on screen	sim fixed base desktop	11	reduced perceptual demands with haptic lateral guidance
Easa and He (2006)	self-paced onset, fixed open duration (.5 s), total field of view	sim fixed base desktop	15	effects of 3D highway alignments on visual demand
Chen and Milgram (2011), experiment I–III	self-paced onset, fixed open duration (.25 s, .5 s, 1.0 s, 2.0 s, 4.0 s), total field of view	test track	12	how to select open duration in occlusion experiments
Chen and Milgram (2011), experiment IV	self-paced onset, fixed open duration (.25 s, .5 s, 1.0 s, 2.0 s, 4.0 s), total field of view	sim fixed base	12	how to select open duration in occlusion experiments
Chen and Milgram (2013)	self-paced onset, fixed open duration (1.0 s), total field of view	closed motorway	6	prediction of lane deviation depending on occlusion duration
Mars et al. (2014)	self-paced onset, fixed open duration (1.0 s), total field on screen	sim fixed base	22	changes in visual demand with the amount of haptic lateral guidance
Kujala et al. (2016)	self-paced onset, fixed open duration (.5 s), total field on screen	sim moving base	97	correspondence between information density and occlusion distance
Pekkanen et al. (2017)	self-paced onset, fixed open duration (.3 s), central field on screen	sim fixed base desktop	18	time headway selection varies as a function of driver capability to maintain a preferred task difficulty
Pekkanen et al. (2018) ^a^	self-paced onset, fixed open duration (.3 s), central field on screen/windshield	sim fixed base VR + test track	40	visual sampling is driven by uncertainty

*Note*. ^a^Eye- tracking was used simultaneously with occlusion.
